# Overcoming Bottlenecks for Metabolic Engineering of Sesquiterpene Production in Tomato Fruits

**DOI:** 10.3389/fpls.2021.691754

**Published:** 2021-06-17

**Authors:** Michael Gutensohn, Laura K. Henry, Scott A. Gentry, Joseph H. Lynch, Thuong T. H. Nguyen, Eran Pichersky, Natalia Dudareva

**Affiliations:** ^1^Division of Plant and Soil Sciences, West Virginia University, Morgantown, WV, United States; ^2^Department of Biochemistry, Purdue University, West Lafayette, IN, United States; ^3^Purdue Center for Plant Biology, Purdue University, West Lafayette, IN, United States; ^4^Department of Molecular, Cellular and Developmental Biology, University of Michigan, Ann Arbor, MI, United States; ^5^Department of Horticulture and Landscape Architecture, Purdue University, West Lafayette, IN, United States

**Keywords:** sesquiterpenes, monoterpenes, metabolic engineering, tomato fruit, nerolidol/linalool synthase, MEP pathway, MVA pathway, isopentenyl phosphate kinase

## Abstract

Terpenoids are a large and diverse class of plant metabolites that also includes volatile mono- and sesquiterpenes which are involved in biotic interactions of plants. Due to the limited natural availability of these terpenes and the tight regulation of their biosynthesis, there is strong interest to introduce or enhance their production in crop plants by metabolic engineering for agricultural, pharmaceutical and industrial applications. While engineering of monoterpenes has been quite successful, expression of sesquiterpene synthases in engineered plants frequently resulted in production of only minor amounts of sesquiterpenes. To identify bottlenecks for sesquiterpene engineering in plants, we have used two nearly identical terpene synthases, snapdragon (*Antirrhinum majus*) nerolidol/linalool synthase-1 and -2 (AmNES/LIS-1/-2), that are localized in the cytosol and plastids, respectively. Since these two bifunctional terpene synthases have very similar catalytic properties with geranyl diphosphate (GPP) and farnesyl diphosphate (FPP), their expression in target tissues allows indirect determination of the availability of these substrates in both subcellular compartments. Both terpene synthases were expressed under control of the ripening specific *PG* promoter in tomato fruits, which are characterized by a highly active terpenoid metabolism providing precursors for carotenoid biosynthesis. As *AmNES/LIS-2* fruits produced the monoterpene linalool, *AmNES/LIS-1* fruits were found to exclusively produce the sesquiterpene nerolidol. While nerolidol emission in *AmNES/LIS-1* fruits was 60- to 584-fold lower compared to linalool emission in *AmNES/LIS-2* fruits, accumulation of nerolidol-glucosides in *AmNES/LIS-1* fruits was 4- to 14-fold lower than that of linalool-glucosides in *AmNES/LIS-2* fruits. These results suggest that only a relatively small pool of FPP is available for sesquiterpene formation in the cytosol. To potentially overcome limitations in sesquiterpene production, we transiently co-expressed the key pathway-enzymes hydroxymethylglutaryl-CoA reductase (HMGR) and 1-deoxy-D-xylulose 5-phosphate synthase (DXS), as well as the regulator isopentenyl phosphate kinase (IPK). While HMGR and IPK expression increased metabolic flux toward nerolidol formation 5.7- and 2.9-fold, respectively, DXS expression only resulted in a 2.5-fold increase.

## Introduction

Plants produce volatile organic compounds (VOCs) in leaves, flowers and fruits as well as roots and release them into the atmosphere and soil. Emitted VOCs play key roles in attracting pollinators and seed dispersers, directly or indirectly protecting plants against herbivores and pathogens, and mediating plant-plant communication (Dudareva et al., 2013). Based on their biosynthetic origin, plant VOCs are divided into several classes including phenylpropanoids/benzenoids, fatty acid derivatives, amino acid derivatives, and terpenoids. Terpenoids represent one of the largest and most diverse classes of plant metabolites that are involved in various basic physiological processes including photosynthesis, respiration, growth, and development (Vranová et al., 2013; Pichersky and Raguso, 2016). In addition to essential compounds such as sterols, carotenoids, chlorophylls, quinones, and the hormones gibberellins, strigolactones, abscisic acid and brassinosteroids, this metabolite class also includes the volatile mono- and sesquiterpenes that play important roles in biotic interactions of plants.

**Figure 1:**
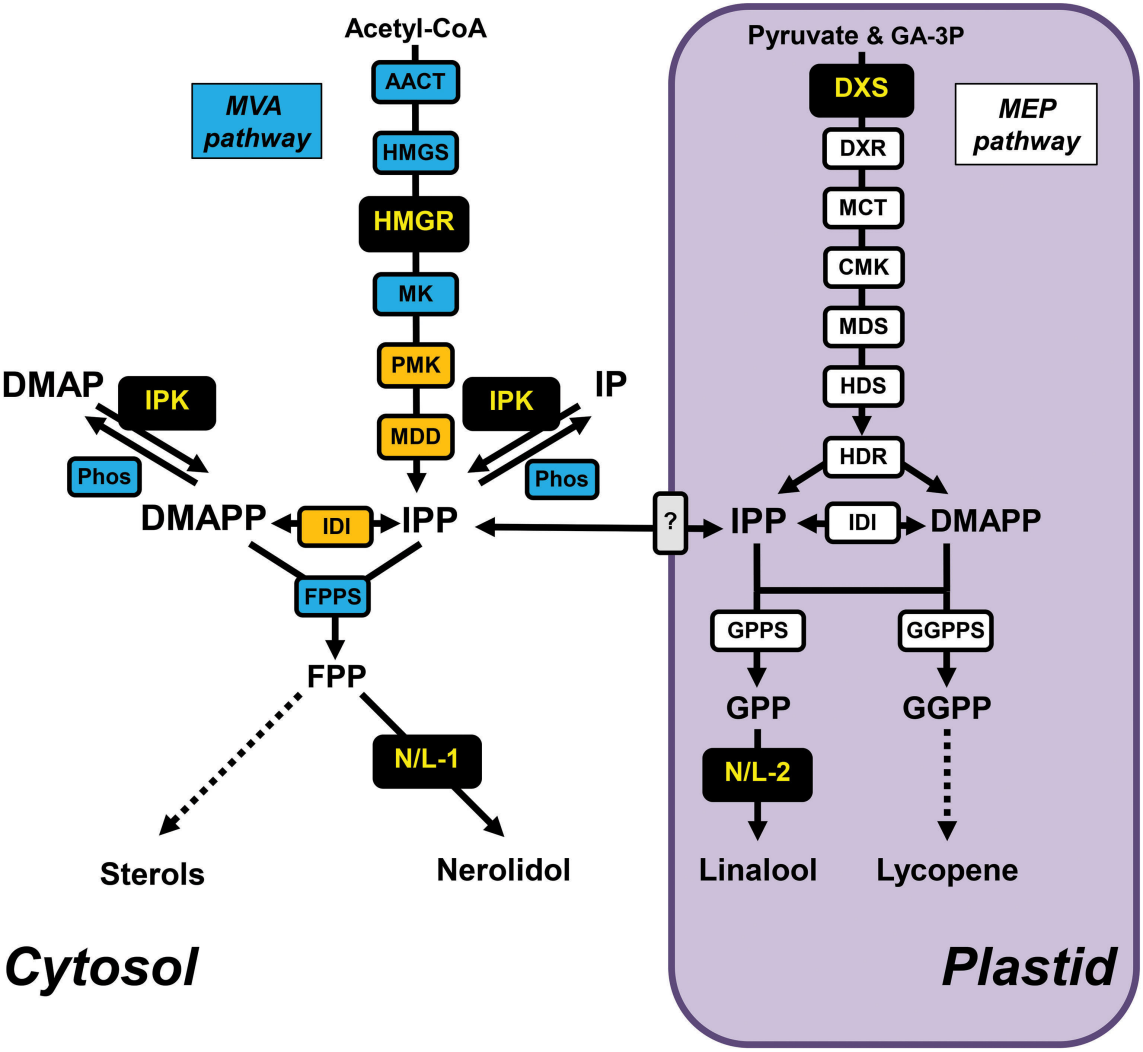
Terpenoid metabolic network in tomato fruits and position of engineered enzymes. Cytosolic and plastidic (highlighted in purple) terpenoid metabolic pathways involved in the biosynthesis of sterols and lycopene in tomato fruits with individual enzymes depicted as boxes. The cytosolic MVA pathway enzymes, prenyl transferase, and Nudix hydrolase are highlighted in blue, peroxisomal MVA pathway enzymes are highlighted in orange, the plastidic MEP pathway enzymes and prenyl transferases are highlighted in white. The unknown transporter(s) involved in IPP exchange across the plastid envelope membranes are shown in gray. The enzymes that have been overexpressed in tomato fruits for metabolic engineering are highlighted in black, including the *Antirrhinum majus* terpene synthases AmNES/LIS-1 (N/L-1) and AmNES/LIS-2 (N/L-2) catalyzing the formation of nerolidol and linalool, respectively. Dashed lines indicate multiple enzymatic steps. AACT, acetoacetyl-CoA thiolase; CMK, 4-(cytidine 5'-diphospho)-2-*C*-methyl-D-erythritol kinase; DMAP, dimethylallyl phosphate; DMAPP, dimethylallyl diphosphate; DXR, 1-deoxy-D-xylulose 5-phosphate reductoisomerase; DXS, 1-deoxy-D-xylulose 5-phosphate synthase; FPP, farnesyl diphosphate; FPPS, farnesyl diphosphate synthase; GA-3P, D-glyceraldehyde 3-phosphate; GPP, geranyl diphosphate; GPPS, geranyl diphosphate synthase; GGPP, geranylgeranyl diphosphate; GGPPS, geranylgeranyl diphosphate synthase; HDR, (*E*)-4-hydroxy-3-methylbut-2-enyl diphosphate reductase; HDS, (*E*)-4-hydroxy-3-methylbut-2-enyl diphosphate synthase; HMGR, 3-hydroxy-3-methylglutaryl-CoA reductase; HMGS, 3-hydroxy-3-methylglutaryl-CoA synthase; IDI, isopentenyl diphosphate isomerase; IPK, isopentenyl phosphate kinase; IP, isopentenyl phosphate; IPP, isopentenyl diphosphate; MCT, 2-*C*-methyl-D-erythritol 4-phosphate cytidylyltransferase; MDD, mevalonate diphosphate decarboxylase; MDS, 2-*C*-methyl-D-erythritol 2,4-cyclodiphosphate synthase; MK, mevalonate kinase; Phos, Nudix hydrolase(s); PMK, phosphomevalonate kinase.

All terpenoids originate from the C5 building block isomers isopentenyl diphosphate (IPP) and dimethylallyl diphosphate (DMAPP), which in plants are synthesized by two alternative pathways that are localized in different subcellular compartments (Ashour et al., 2010; Hemmerlin et al., 2012). The mevalonic acid (MVA) pathway is localized in the cytosol and partially in peroxisomes (Figure 1), while the methylerythritol phosphate (MEP) pathway operates in plastids. Although these pathways act independently, there is substantial evidence for metabolic crosstalk between them (Hemmerlin et al., 2012; Vranová et al., 2013) with IPP and DMAPP exchange potentially occurring in both directions via yet unidentified transporter(s) in the plastid envelope membranes (Soler et al., 1993; Bick and Lange, 2003; Flügge and Gao, 2005). IPP and DMAPP generated by both pathways are subsequently utilized by prenyltransferases to form larger prenyl diphosphate intermediates that ultimately serve as precursors for the downstream biosynthesis of terpenoid compounds. Farnesyl diphosphate (FPP) is formed in the cytosol by FPP synthase (FPPS), while geranyl diphosphate (GPP) and geranylgeranyl diphosphate (GGPP) are produced in plastids by GPP synthase (GPPS) and GGPP synthase (GGPPS), respectively (Figure 1). While cytosolic FPP serves as a precursor for sterols and brassinosteroids, plastidic GGPP is utilized for chlorophyll, carotenoid, strigolactone, abscisic acid and gibberellin biosynthesis. In addition, FPP is used by cytosolic sesquiterpene synthases, whereas plastidic monoterpene synthases use GPP as substrate. Recently a new regulatory machinery was identified in plants (Figure 1) that is composed of isopentenyl phosphate kinase (IPK) and a subset of Nudix superfamily hydrolases (Henry et al., 2015, 2018). These two enzymes appear to modulate the equilibrium between isopentenyl phosphate (IP)/dimethylallyl phosphate (DMAP) and IPP/DMAPP, and in consequence the metabolic flux toward downstream terpenoid products.

Due to their roles in attracting beneficial insects, defending against pests and pathogens, and contributing to the aromas of fruits and other edible parts of plants, volatile mono- and sesquiterpenes are increasingly considered as valuable agronomic traits. In addition, these plant-derived terpenes are widely used by humans as flavors, fragrances, preservatives, biofuels and pharmaceuticals (Ajikumar et al., 2008; Immethun et al., 2013; Tippmann et al., 2013). However, because of the involvement of volatile monoterpenes and sesquiterpenes in specialized biological processes, their biosynthesis in plants is often restricted to specific tissues and developmental stages or induced by environmental stimuli (summarized in Gutensohn and Dudareva, 2016). Moreover, the lack of attention to terpene volatiles as traits in conventional crop breeding programs has led to the loss of such traits and as a result current varieties of many crops produce little to no terpene compounds (Köllner et al., 2008).

Metabolic engineering represents an efficient approach to either increase or modify terpene formation in naturally producing plant species, or to introduce a terpene biosynthetic pathway into a plant species naturally devoid of a compound of interest. Over the last decade numerous attempts have been made to engineer the formation of mono- and sequiterpene compounds in various plant species and tissues (summarized in Lange and Ahkami, 2013; Vickers et al., 2014). While engineering of monoterpenes in plants has been successful, the overexpression of sesquiterpene synthases in transgenic plants frequently resulted in the production of only small amounts of sesquiterpenes. Indeed, when a number of diverse sesquiterpene synthases, including a fungal trichodiene synthase (Hohn and Ohlrogge, 1991), germacrene A synthase from chicory (Aharoni et al., 2003), amorpha-4,11-diene synthase from *Artemisia annua* (Wallaart et al., 2001; Wu et al., 2006) and patchoulol synthase from *Pogostemon cablin* (Wu et al., 2006), were expressed in Arabidopsis or tobacco plants only trace to low levels of the target sesquiterpene products were observed.

Likewise, the overexpression of the cytosolic maize terpene synthase 10 (TPS10) (Schnee et al., 2006) or mint (*E*)-β-farnesene synthase (Beale et al., 2006) in Arabidopsis and oregano (*E*)-β-caryophyllene synthase in maize (Degenhardt et al., 2009) resulted in the formation of only moderate levels of the respective sesquiterpenes, although these levels were sufficient to elicit potent effects on the behavior of herbivores, predators, and parasitoids. In contrast, the expression of α-zingiberene synthase (ZIS), a multiproduct sesquiterpene synthase from *Ocimum basilicum*, in tomato fruits led to the accumulation of a significant amount of sesquiterpene products (Davidovich-Rikanati et al., 2008). However, the total amount of sesquiterpenes produced in these *ZIS* tomato fruits was ~3.5-fold lower than the total amount of monoterpenes that accumulated in tomato fruits expressing *Ocimum basilicum* geraniol synthase (GES) (Davidovich-Rikanati et al., 2007) even though both terpene synthases were expressed under the control of the same fruit ripening specific promoter. Together, these results suggest that often FPP, the immediate precursor for all sesquiterpenes, is not readily available for catalysis by the introduced sesquiterpene synthases, although FPP is expected to be produced efficiently in the cytosol to support the demand for sterol biosynthesis in many plant tissues.

The internal concentrations of FPP and GPP in the cytosolic and plastid compartments of plant tissues and organs involved in terpene biosynthesis have been proven extremely difficult to measure. Previous attempts (McCaskill and Croteau, 1993; Nogues et al., 2006) have only succeeded in ascertaining that the concentrations of these compounds are relatively low, which could be underestimated due to their high instability, and sensitivity to enzymatic and non-enzymatic hydrolysis. Alternative approaches via genetic engineering in which a heterologous terpene synthase (TPS) gene was introduced under a strong promoter into a host plant also provided some indirect information on the availability of these substrates. However, due to the different affinity of any single terpene synthase toward FPP and GPP, results from such experiments are difficult to interpret, even when the enzyme is directed to both the cytosol and the plastid in separate experiments. A better comparison could be made by using two enzymes that have similar kinetic properties for the prenyl diphosphate substrates, one directed to the cytosol and the other to plastids.

While for many TPSs their sesquiterpene-forming activities significantly exceed their monoterpene-forming activities or vice versa, in this study we took advantage of two closely related snapdragon terpene synthases, AmNES/LIS-1 and AmNES/LIS-2 (nerolidol/linalool synthase-1 and -2), which exhibit very similar kinetic properties, to indirectly determine the pool sizes of GPP and FPP precursors in the cytosolic and plastidic compartments. These two TPSs share 95% amino acid sequence identity and were shown to accept both GPP and FPP substrates with similar catalytic efficiencies (*k*_*cat*_/*K*_*m*_ ratio) of ~2.8 mM^−1^s^−1^ and ~4.1 mM^−1^s^−1^, respectively (Nagegowda et al., 2008). The difference between these two enzymes is that AmNES/LIS-1 has no N-terminal transit peptide and is localized in the cytosol, while AmNES/LIS-2 has a transit peptide directing it to plastids.

Here, we expressed the cytosolic AmNES/LIS-1 and plastidic AmNES/LIS-2 in ripening tomato fruits to better understand the availability of each substrate, FPP and GPP, in both compartments. Tomato fruit is a good model system for these experiments since the terpenoid metabolic network, in particular the plastidic MEP pathway, is active providing precursors for the accumulation of lycopene and other carotenoids (Lawrence et al., 1997; Lois et al., 2000; Botella-Pavia et al., 2004; Paetzold et al., 2010), while only very little mono- and sesquiterpenes are formed in ripening tomato fruit (Zhou and Pichersky, 2020). There is ample evidence that the precursors of terpenoids, namely IPP and DMAPP, can be shuttled between plastids and cytosol. Thus, an analysis of FPP and GPP availability for terpene biosynthesis has to consider that the substrate depletion by a TPS introduced in one compartment, could potentially be ameliorated by transport from the other compartment. To evaluate the effect of increased flux through the cytosolic MVA and plastidic MEP pathways on the availability of FPP and GPP substrates in the two cellular compartments, we also co-expressed the key pathway-enzymes 3-hydroxy-3-methyl-glutaryl-CoA reductase (HMGR) and 1-deoxy-D-xylulose 5-phosphate synthase (DXS), respectively, in *AmNES/LIS-1* and *-2* tomato fruits. In addition, we tested whether the recently identified key regulator isopentenyl phosphate kinase can further enhance the metabolic flux toward cytosolic sesquiterpene formation in *AmNES/LIS-1* tomato fruits.

## Materials and Methods

### Plant Material and Growth Conditions

Tomato (*Solanum lycopersicum*) line MP1 (Barg et al., 1997) was used for generation of transgenic plants and as control for all analyses. Plants were grown under a 14 h photoperiod in standard greenhouse conditions. MP1 plants were grown from seeds, while all transgenic lines (*AmNES/LIS-1, AmNES/LIS-2)* were propagated by re-rooting of cuttings.

### Vector Construction and Stable Plant Transformation

The coding regions of *Antirrhinum majus AmNES/LIS-1* and *AmNES/LIS-2* (Nagegowda et al., 2008) were cloned into the binary vector pBIN19 carrying the tomato *PG* promoter and terminator, and the kanamycin-resistance marker *NPTII* driven by the *CaMV 35S* promoter (Nicholass et al., 1995). Transgenic tomato plants were generated via *Agrobacterium tumefaciens* (strain c58C1/pMP90) (Koncz and Schell, 1986) mediated transformation as described (McCormick et al., 1986). Transgenic plants rooted on kanamycin (100 mg L^−1^) selection medium were transferred to soil and adapted to greenhouse conditions.

### Initial Screening of Transgenic Plants

Genomic DNA was extracted from tomato leaves as described previously (Ausubel et al., 1994). Tomato lines were screened for the presence of transgenes by PCR on genomic DNA using gene-specific primers for *AmNES/LIS-1* and *-2* (Supplementary Table 1). Confirmed transgenic *AmNES/LIS-1* and *-2* lines were further analyzed for nerolidol and linalool formation, respectively, in ripe tomato fruits using a solid-phase microextraction (SPME) system and gas chromatography-mass spectrometry (GC-MS).

### RNA Isolation and qRT-PCR Analysis

Total RNA was isolated from tomato fruits as described (Eggermont et al., 1996). For quantitative real-time PCR (qRT-PCR) analysis, RNA was pretreated with RNase-free DNase (Promega) and cDNA was synthesized using Reverse Transcriptase (Superscript II, Invitrogene). Gene specific primers were designed using the PrimerExpress software (Applied Biosystems) and showed more than 90% efficiency at final concentrations of 500 nM (Supplementary Table 1). qRT-PCR reactions were performed and transcript levels were determined as previously described (Orlova et al., 2009; Gutensohn et al., 2013) using the StepOne Real-Time PCR system (Applied Biosystems). For the absolute quantification of *AmNES/LIS-1* and *-2* transcript levels by qRT-PCR, respective cDNA fragments were purified, diluted from 2 ng/ml to 3.2 pg/ml, and used as templates to obtain standard curves in qRT-PCR with gene specific primers (Supplementary Table 1). Based on standard curves, absolute quantities of individual transcripts were calculated and expressed as percentage of total mRNA. Each data point represents an average of at least three independent biological samples with three technical replicates for each sample.

### Collection, Extraction, and Analysis of Terpenes From Tomato Fruits

Volatile terpenes emitted from ripe tomato fruits (Breaker +10 days) were collected for 24 h using a closed-loop stripping method as described previously (Dudareva et al., 2005; Gutensohn et al., 2013; Wang et al., 2020). For analysis of internal terpene pools pericarp of ripe tomato fruits was extracted with methyl *tert*-butyl ether (MTBE) as described previously (Davidovich-Rikanati et al., 2007; Gutensohn et al., 2013). Terpene glucosides were extracted with methanol and enzymatically hydrolyzed to release the terpene aglycone as described previously (Boachon et al., 2019). Emitted and extracted volatiles were analyzed by GC-MS as described (Dudareva et al., 2005) using naphthalene as internal standard. Nerolidol and linalool standards were used to determine response factors, which were applied for the quantification.

### Transient Transformation of Tomato Fruits

Open reading frames of *AtHMGR1* (At1g76490), *AtDXS* (At4g15560), and *AtIPK* (At1g26640) in the pENTR vector were obtained from the Arabidopsis Biological Resource Center (G12571, Gc104921, GC104863, respectively) and transferred into the binary vector pB2GW7 under control of the *CaMV 35S* promoter using the Gateway LR Clonase II (Invitrogen). The resulting binary vectors were introduced into *A. tumefaciens* (strain LBA 4404) and those subsequently used for transient transformation of tomato fruits as described (Orzaez et al., 2006). *Agrobacterium* suspensions were injected into ripening fruits (Breaker +3 days) of transgenic *AmNES/LIS-1* and *AmNES/LIS-2* lines using a syringe with hypodermic needle. After 7 days ripe fruits (Breaker +10 days) were harvested, and used for collection and analysis of emitted terpenes as described above. As control *AmNES/LIS-1* and *-2* fruits were injected with *Agrobacterium* carrying the empty pB2GW7 vector and subsequently analyzed in the same way.

## Results

### Overexpression of *AmNES/LIS-1* and *-2* in Tomato Fruits Results in Different Levels of Nerolidol and Linalool Formation

**Figure 2:**
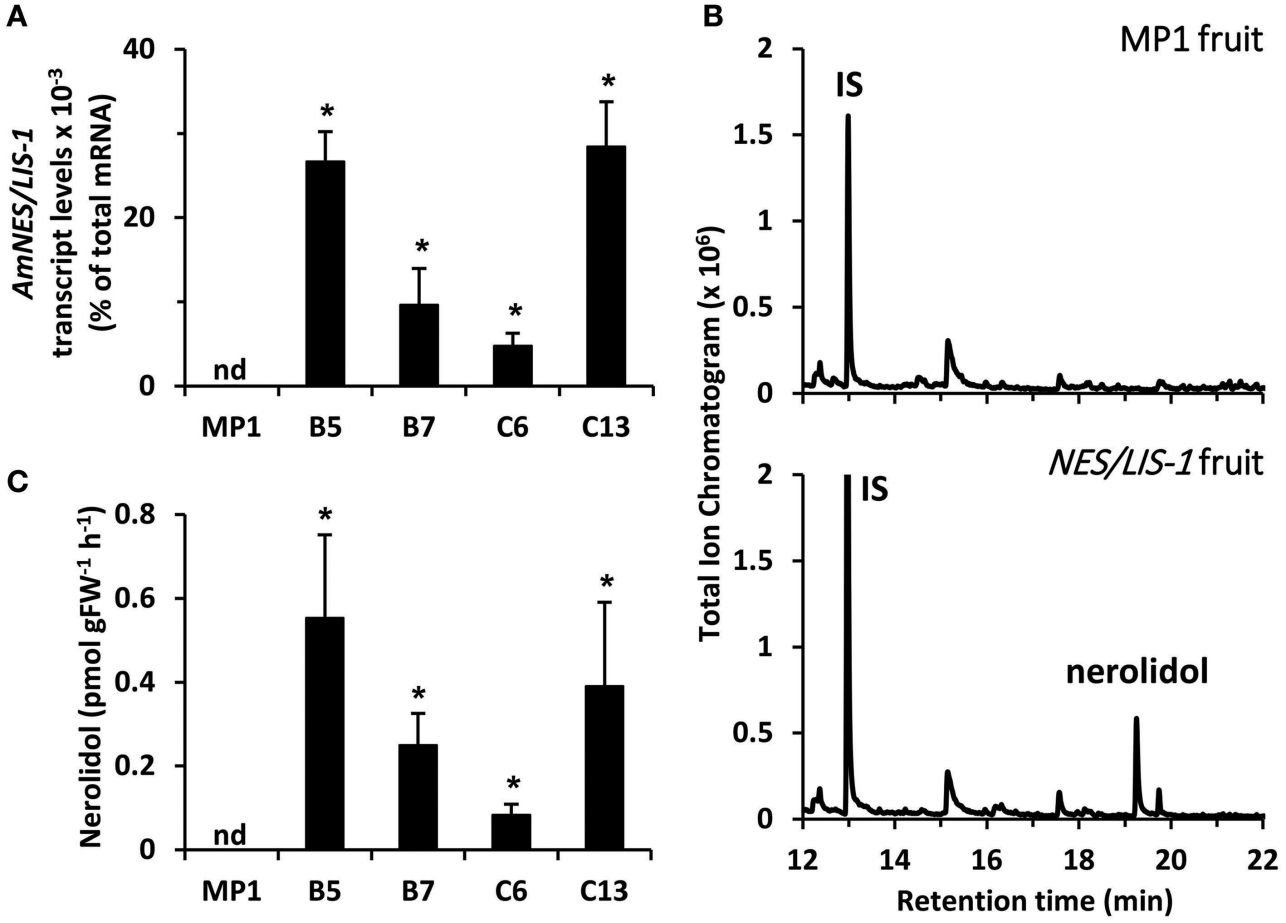
Profiling of transgenic tomato lines overexpressing *AmNES/LIS-1* under control of the *PG* promoter. **(A)** Transcript levels of *AmNES/LIS-1* in fruits (Br + 3 stage) of MP1 control and *AmNES/LIS-1* lines B5, B7, C6, and C13 determined by qRT-PCR (means ± SEM, *n* = 3 biological replicates). **(B)** Profiling of terpenes collected as emitted volatiles from ripe tomato fruits (MP1 control and *AmNES/LIS-1* line B5) and analyzed by GC-MS. IS, internal standard. **(C)** Quantitative changes in amounts of nerolidol collected as emitted volatile from fruits of MP1 and *AmNES/LIS-1* lines. Data are means ± SEM (*n* ≥ 3). *P < 0.05 *AmNES/LIS-1* lines relative to MP1 by Student's *t*-test. FW, fresh weight; nd, not detected.

**Figure 3:**
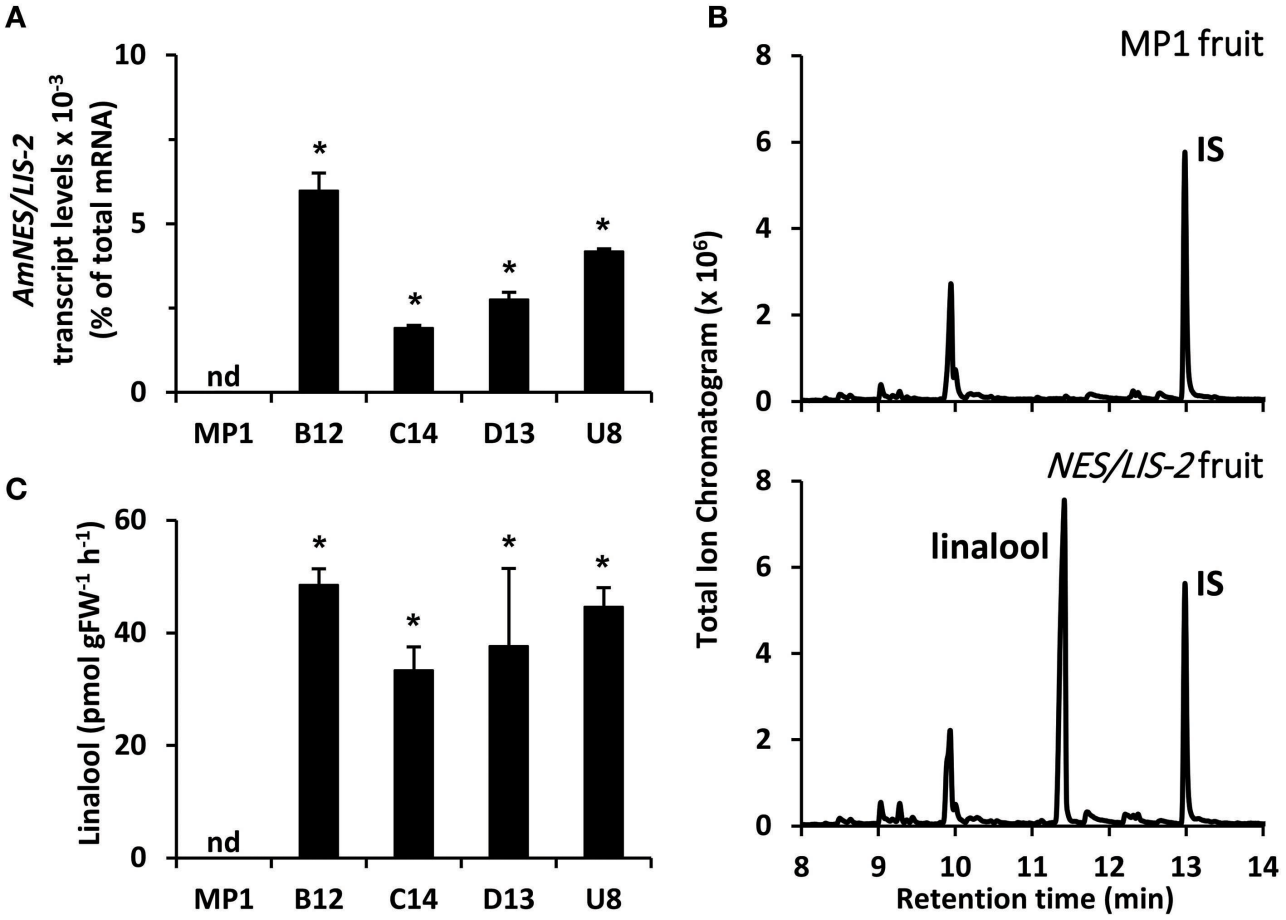
Profiling of transgenic tomato lines overexpressing *AmNES/LIS-2* under control of the *PG* promoter. **(A)** Transcript levels of *AmNES/LIS-2* in fruits (Br + 3 stage) of MP1 control and *AmNES/LIS-2* lines B12, C14, D13, and U8 determined by qRT-PCR (means ± SEM, *n* = 3 biological replicates). **(B)** Profiling of terpenes collected as emitted volatiles from ripe tomato fruits (MP1 control and *AmNES/LIS-2* line U8) and analyzed by GC-MS. IS, internal standard. **(C)** Quantitative changes in amounts of linalool collected as emitted volatile from fruits of MP1 and *AmNES/LIS-2* lines. Data are means ± SEM (*n* ≥ 3). *P < 0.05 *AmNES/LIS-2* lines relative to MP1 by Student's *t*-test. FW, fresh weight; nd, not detected.

To determine the level of available FPP and GPP precursors for sesquiterpene and monoterpene production, respectively, in tomato fruits, we overexpressed the cytosolic AmNES/LIS-1 and plastidic AmNES/LIS-2, the two nearly identical bifunctional terpene synthases from snapdragon (Nagegowda et al., 2008), in tomato plants under control of the fruit ripening specific *PG* promoter (Nicholass et al., 1995). This promoter limits transgene expression to the pericarp of ripening fruit, thus avoiding negative effects on vegetative tissues and general plant development (Orlova et al., 2009). After an initial screening of 29 tomato lines for the presence of the *AmNES/LIS-1* transgene as well as nerolidol formation, four independent lines (B5, B7, C6, and C13) with various *AmNES/LIS-1* expression levels, as determined by quantitative real-time PCR (Figure 2A), were chosen for further characterization. Likewise, four independent *AmNES/LIS-2* overexpressing lines (B12, C14, D13, and U8) with different expression levels (Figure 3A) were selected for detailed analysis from 56 transgenic tomato lines after an initial screening for the presence of the *AmNES/LIS-2* transgene and linalool formation.

To examine sesquiterpene and monoterpene formation upon *AmNES/LIS-1* and *AmNES/LIS-2* overexpression, respectively, emitted volatiles were collected from ripe tomato fruits (Br +10 stage) and analyzed by gas chromatography-mass spectrometry (GC-MS). The only difference in the volatile profile between transgenic lines and control was the formation of the sesquiterpene nerolidol and the monoterpene linalool in *AmNES/LIS-1* (Figure 2B) and *AmNES/LIS-2* (Figure 3B) transgenic fruits, respectively, which were absent in the MP1 control fruits (Figures 2B, 3B). The amounts of nerolidol emitted from *AmNES/LIS-1* fruits positively correlated with the *AmNES/LIS-1* expression level in the different transgenic lines (Figures 2A,C). A similar positive correlation between the amounts of linalool emitted from fruits and the respective *AmNES/LIS-2* transcript levels was observed in the different transgenic *AmNES/LIS-2* lines (Figures 3A,C). No formation of linalool was observed in *AmNES/LIS-1* fruits and likewise *AmNES/LIS-2* fruits did not produce nerolidol (Supplementary Figure 1). The most remarkable outcome of the characterization of these transgenic *AmNES/LIS-1* and *AmNES/LIS-2* fruits was that despite the almost identical kinetic properties of the two expressed terpene synthases (Nagegowda et al., 2008), and similar or higher expression levels of *AmNES/LIS-1* (4.78 ± 1.50 to 28.43 ± 5.31 × 10^−3^% of total mRNA) relative to that of *AmNES/LIS-2* (1.90 ± 0.08 to 5.97 ± 0.52 × 10^−3^% of total mRNA) (Figures 2A, 3A), nerolidol emission in *AmNES/LIS-1* fruits was 60- to 584-fold lower than linalool emission in *AmNES/LIS-2* fruits (Figures 2C, 3C).

### *AmNES/LIS-1* Tomato Fruits Accumulate Internal Pools of Nerolidol Derivatives

**Figure 4:**
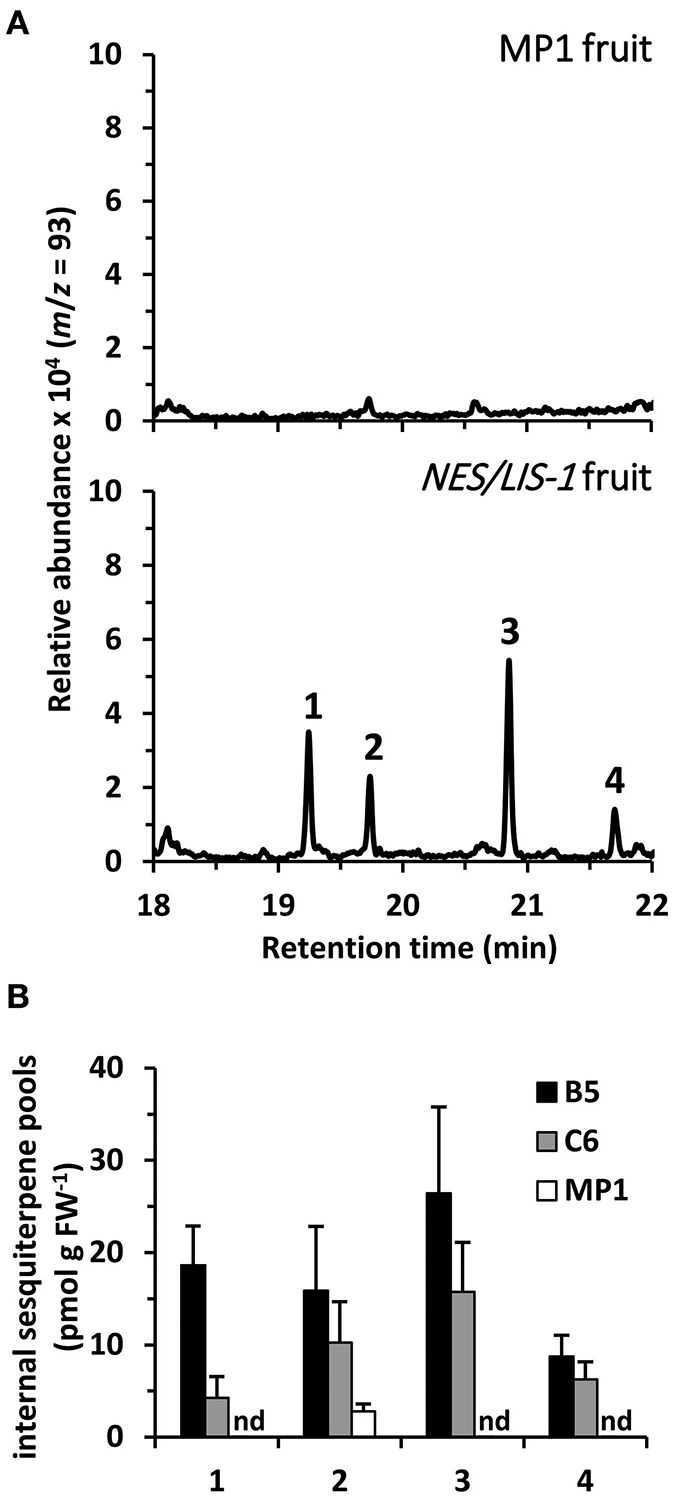
Metabolic profiling of internal sesquiterpene pools in *AmNES/LIS-1* fruits. **(A)** Terpenes were directly extracted from ripe fruits of MP1 control and *AmNES/LIS-1* transgenic tomato (line B5). Extracts were analyzed by GC-MS and traces obtained for *m/z* = 93 (characteristic for terpenes) are shown. Based on their mass spectra and retention time the following compounds were identified: 1, nerolidol; 2, unknown 1; 3, unknown 2; 4, nerolidyl acetate. **(B)** Quantitative analysis of nerolidol and its derivatives extracted from fruits of the *AmNES/LIS-1* lines B5 and C6, and the MP1 control. Data are means ± SEM (*n* ≥ 3). FW, fresh weight; nd, not detected.

Earlier metabolic engineering attempts to produce volatile terpenes in different plant tissues, including tomato fruits, resulted in formation not only of the desired product but also of other terpenes, which are derived from the primary product by the action of endogenous plant enzymes (Lewinsohn et al., 2001; Lücker et al., 2001; Davidovich-Rikanati et al., 2007). These product modifications often affect the volatility of the derived compounds and result in their accumulation inside the tissue. Thus, as a first step to further evaluate the underlying mechanisms causing the low levels of nerolidol emission in *AmNES/LIS-1* fruits relative to the linalool emission in *AmNES/LIS-2* fruits, we analyzed internal terpene pools in *AmNES/LIS-1* fruits (Figure 4). GC-MS analysis of terpenes extracted from ripe tomato fruits with methyl *tert*-butyl ether revealed four different terpene compounds in *AmNES/LIS-1* fruits that were absent in the MP1 control fruits (Figure 4A). Besides nerolidol, the transgenic fruits accumulated nerolidyl acetate as well as two unknown terpene compounds (mass spectra shown in Supplementary Figure 2). Comparative analysis of the internal pools of nerolidol and its three derivatives in the lines B5 and C6 with high and low *AmNES/LIS-1* expression levels, respectively (Figure 2A), revealed a trend toward accumulating larger amounts of these terpene compounds in line B5 vs. line C6 (Figure 4B). However, only the internal nerolidol pool was statistically higher in line B5. While the metabolic profiling suggests that some of the nerolidol produced in transgenic *AmNES/LIS-1* fruits is indeed converted to other compounds by endogenous enzymes, such as acetyl transferases, the accumulated compounds (in the pmol/g FW range) cannot explain the roughly 100-fold difference in nerolidol vs. linalool emission observed in *AmNES/LIS-1* and *AmNES/LIS-2* fruits, respectively (Figures 2C, 3C).

### *AmNES/LIS-1* and *-2* Tomato Fruits Accumulate Glycosylated Terpenes

**Figure 5:**
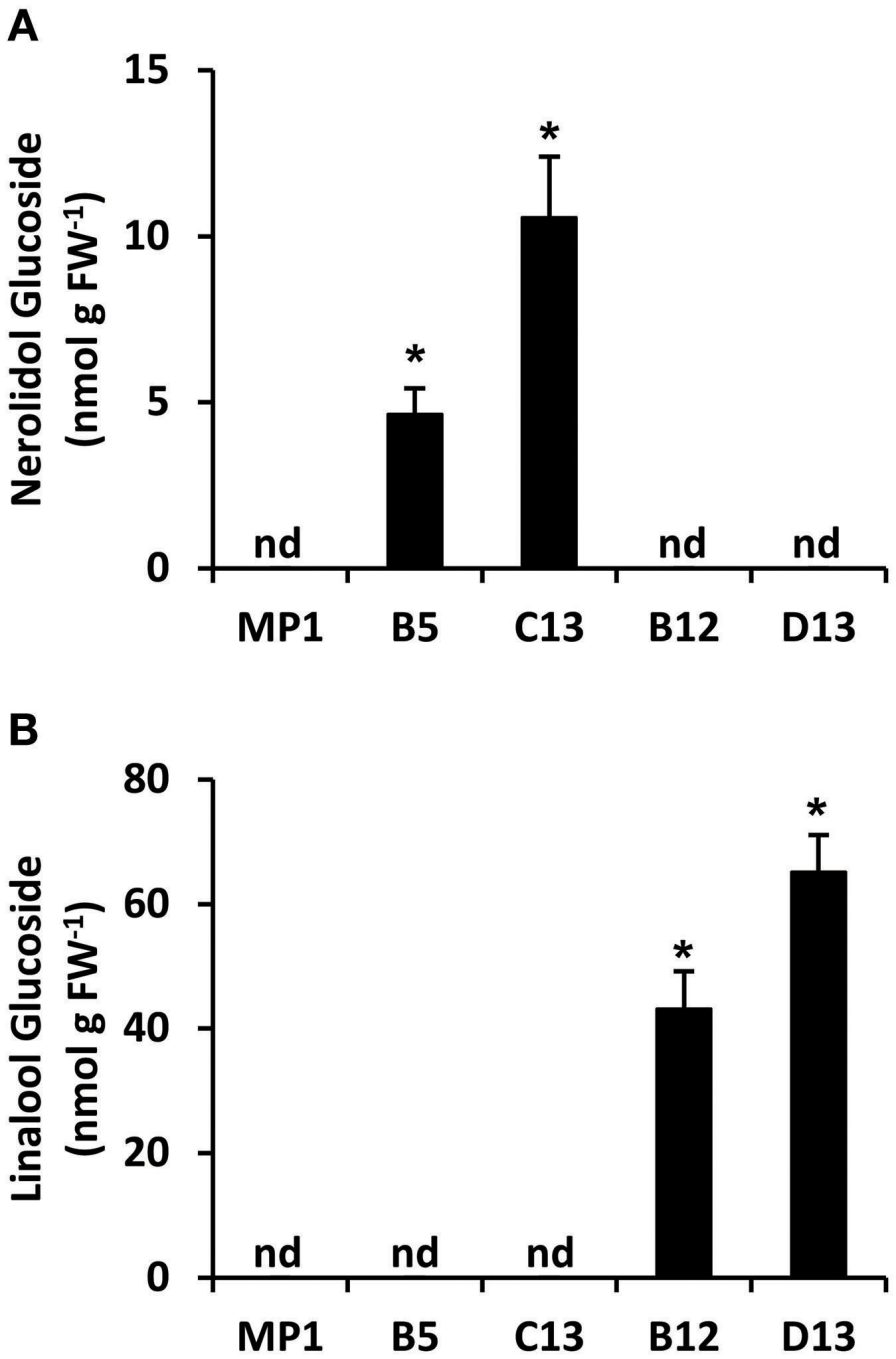
Metabolic profiling of terpene glucosides in *AmNES/LIS-1* (lines B5 and C13) and *AmNES/LIS-2* (lines B12 and D13) fruits. Glucosides were extracted from ripe fruits of MP1 control and representative transgenic lines, then enzymatically hydrolyzed. The released nerolidol **(A)** and linalool **(B)** aglycones were analyzed by GC-MS. Data are means ± SEM (*n* = 3). *P < 0.05 *AmNES/LIS-1/-2* lines relative to the MP1 control by Student's *t*-test. FW, fresh weight; nd, not detected.

Terpenes with hydroxyl functional groups, such as nerolidol and linalool, can be glycosylated by non-specific glucotransferases to form non-volatile, inert end products. To test whether the apparent discrepancy between production of nerolidol and linalool in *AmNES/LIS-1* and *-2* fruits, respectively, may be the consequence of an unequal flux toward respective glucoside derivatives, we analyzed terpene glucoside accumulation in ripe tomato fruits (Br + 10 stage) (Figure 5) from the MP1 control, and representative *AmNES/LIS-1* and *-2* lines showing high transgene expression. Nerolidol-glucosides were detected only in fruits of the *AmNES/LIS-1* lines, and linalool-glucosides were only present in fruits of the *AmNES/LIS-2* lines (Figures 5A,B). Remarkably, a substantial part of both, the nerolidol and linalool formed in *AmNES/LIS-1* and *-2* lines, respectively, appears to be converted to glucosides and accumulated as internal pools (in the nmol/g FW range) in these fruits. However, consistent with the difference in emission of the volatile free nerolidol and linalool (Figures 2C, 3C), the amount of nerolidol-glucosides in the *AmNES/LIS-1* lines was 4- to 14-fold lower than the amount of linalool-glucosides in the *AmNES/LIS-2* lines (Figure 5). Therefore, these results further confirm a significant difference in the potential to produce sesquiterpenes and monoterpenes in tomato fruits.

### Co-expression of *HMGR* and *DXS* in *AmNES/LIS-1* and *-2* Fruits Has Different Effects on Nerolidol and Linalool Formation

**Figure 6:**
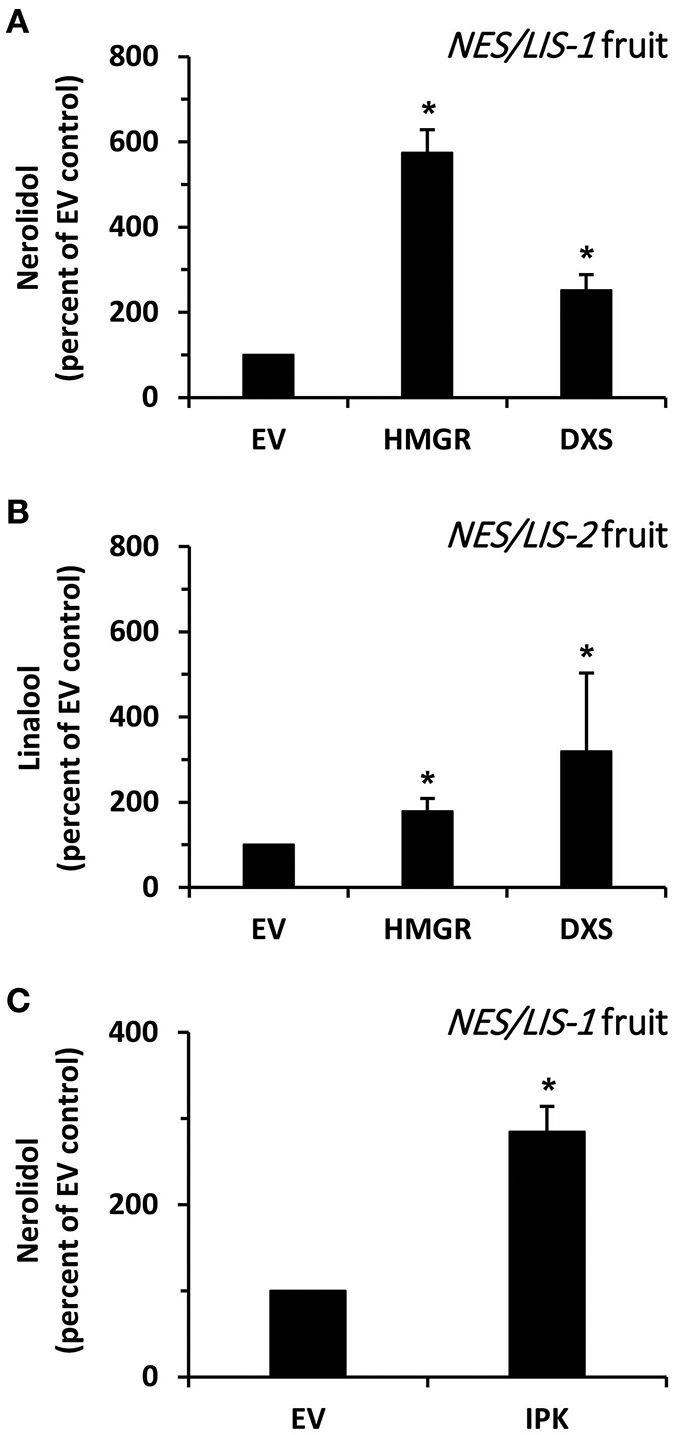
The effect of HMGR, DXS, and IPK overexpression on nerolidol formation in *AmNES/LIS-1* fruits. **(A)** Quantitative changes in amounts of nerolidol emitted from *AmNES/LIS-1* fruits (line C13) upon transient overexpression of *AtHMGR* (HMGR) and *AtDXS* (DXS). Nerolidol contents are presented as percentage of the nerolidol content in *AmNES/LIS-1* fruits injected with *Agrobacterium* carrying the empty pB2GW7 vector (EV), set as 100%. **(B)** Quantitative changes in amounts of linalool emitted from *AmNES/LIS-2* fruits (line U8) upon transient overexpression of *AtHMGR* (HMGR) and *AtDXS* (DXS). Linalool contents are presented as percentage of the linalool content in *AmNES/LIS-2* fruits injected with *Agrobacterium* carrying the empty pB2GW7 vector (EV), set as 100%. **(C)** Quantitative changes in amounts of nerolidol emitted from *AmNES/LIS-1* fruits upon transient overexpression of *AtIPK* (IPK). The nerolidol content is presented as percentage of the nerolidol content in *AmNES/LIS-1* fruits injected with *Agrobacterium* carrying the empty pB2GW7 vector (EV), set as 100%. Data are means ± SEM (*n* ≥ 3). *P < 0.05 *AmNES/LIS-1/-2* lines relative to the EV controls by Student's *t*-test.

Next, we analyzed whether an increase in the flux through the upstream metabolic pathways providing precursors for the introduced AmNES/LIS-1 can enhance nerolidol production in transgenic tomato fruits. It is generally accepted that HMGR catalyzes the rate limiting step in the MVA pathway (Hemmerlin et al., 2012) (Figure 1). Thus, to increase the level of precursors available for the cytosolic AmNES/LIS-1, we transiently overexpressed *Arabidopsis thaliana AtHMGR1* (Enjuto et al., 1994) in ripening *AmNES/LIS-1* fruits using *Agrobacterium* injection (Gutensohn and Dudareva, 2016). Upon co-expression of *AtHMGR1*, emission of nerolidol was found to be 5.7-fold higher in *AmNES/LIS-1* fruits when compared to fruits injected with *Agrobacterium* carrying the empty vector control (Figure 6A), thus indicating that the metabolic flux through the MVA pathway is indeed limiting in ripening tomato fruits.

To analyze if an increased flux through the plastidic MEP pathway could also provide precursors for the cytosolic AmNES/LIS-1 via metabolic crosstalk between plastids and cytosol, we transiently overexpressed *A. thaliana AtDXS* (Estévez et al., 2000), a key enzyme of the MEP pathway (Figure 1), in ripening *AmNES/LIS-1* fruits by *Agrobacterium* injection. Upon co-expression of *AtDXS* in *AmNES/LIS-1* fruits nerolidol emission was increased 2.5-fold compared to fruits injected with *Agrobacterium* carrying the empty vector control (Figure 6A), suggesting that an enhanced metabolic flux through the MEP pathway also supports cytosolic sesquiterpene formation although not to the same extent as an increased flux through the MVA pathway.

In parallel, we also evaluated the effect of higher metabolic fluxes through the MVA and MEP pathways on the linalool formation by AmNES/LIS-2 introduced into transgenic tomato fruits. Therefore, *AtHMGR1* and *AtDXS* were transiently overexpressed in ripening *AmNES/LIS-2* fruits by *Agrobacterium* injection. Subsequent analysis of emitted volatiles showed that linalool formation was 1.8- and 3.2-fold higher upon co-expression of *AtHMGR1* and *AtDXS*, respectively, in *AmNES/LIS-2* fruits compared to fruits injected with *Agrobacterium* carrying the empty vector control (Figure 6B). These results indicate that increased metabolic fluxes through the MVA and MEP pathway both support monoterpene formation in plastids, although the cytosolic MVA pathway contributes to a lesser extent.

### Co-expression of *IPK* in *AmNES/LIS-1* Fruits Significantly Enhances Nerolidol Formation

Isopentenyl phosphate kinase (IPK) was recently identified as a new member of the plant terpenoid metabolic network (Figure 1) and shown to play an important role in modulating the formation of FPP-derived terpenoid compounds, including sesquiterpenes (Henry et al., 2015). In order to find if IPK could also positively influence the FPP levels for sesquiterpene formation in tomato fruits, we transiently overexpressed *A. thaliana AtIPK* (Henry et al., 2015) in ripening *AmNES/LIS-1* fruits by *Agrobacterium* injection. The analysis of emitted volatiles showed that nerolidol formation was 2.9-fold higher upon *AtIPK* co-expression in *AmNES/LIS-1* fruits compared to control fruits injected with *Agrobacterium* carrying the empty vector control (Figure 6C). This result indicates that overexpression of IPK indeed supports nerolidol formation in engineered *AmNES/LIS-1* fruits by shifting the ratio of IP/DMAP to IPP/DMAPP and thus increasing the pool of precursors available for sesquiterpene formation.

## Discussion

To date, there is an increasing demand to introduce or enhance mono- and sesquiterpene traits in crop plants either to provide more sustainable ways of protecting against pests and pathogens, or to achieve improved and lasting aromas of fruits and other edible plant produce. In addition, crop systems can be used as biotechnological platforms for the large-scale production of terpene compounds desired by industry as flavors, fragrances, pharmaceuticals, and biofuels (Ajikumar et al., 2008; Immethun et al., 2013; Tippmann et al., 2013). Naturally the biosynthesis of monoterpenes and sesquiterpenes in plants is often restricted to specific tissues such as glandular trichomes, internal ducts, and flower organs, and is induced by environmental stimuli like herbivore and pathogen attacks (summarized in Gutensohn and Dudareva, 2016). Thus, efforts to improve endogenous terpene traits are limited to such specialized tissues. In contrast, introduction of new terpene traits in crop plants by metabolic engineering often targets tissues in abundant plant organs such as leaves, fruits and seeds. In either case, it is not sufficient to just introduce or increase the expression of a terpene synthase enzyme catalyzing the formation of the terpene product of interest, but it requires a detailed understanding of the availability of the prenyl diphosphate substrates GPP and FPP in the targeted plant tissue and subcellular compartment.

In this study, we utilized two closely related snapdragon bifunctional terpene synthases AmNES/LIS-1 and AmNES/LIS-2 (Nagegowda et al., 2008) that are localized in the cytosol and plastids, respectively. Since these two TPSs, aside from their different subcellular localization, have similar catalytic efficiencies and both accept GPP as well as FPP, their expression in a target tissue allows to indirectly determine the availability of the prenyl diphosphate substrates in the cytosolic and plastidic compartments. Expression of *AmNES/LIS-1* and *AmNES/LIS-2* in tomato plants under control of the fruit ripening specific *PG* promoter (Figures 2A, 3A), resulted in formation of the sesquiterpene nerolidol (Figure 2B) and the monoterpene linalool (Figure 3B) in transgenic fruits, respectively. Despite the fact that these snapdragon TPSs are capable to use both prenyl diphosphate substrates (Nagegowda et al., 2008), no linalool and nerolidol production was found in *AmNES/LIS-1* and *AmNES/LIS-2* fruits, respectively (Supplementary Figure 1). In particular, the absence of linalool formation in *AmNES/LIS-1* fruits was surprising. Earlier studies (Davidovich-Rikanati et al., 2008; Gutensohn et al., 2013) had demonstrated that expression of the cytosolic bifunctional TPS, *Ocimum basilicum* α-zingiberene synthase (ZIS), in ripening tomato fruits resulted not only in the expected formation of sesquiterpenes driven by the cytosolic FPP, but also some monoterpene formation, suggesting the presence of a small GPP pool that appears to be derived from plastids. However, the contrasting results obtained with *AmNES/LIS-1* and *ZIS* fruits could be explained by the kinetic properties of the introduced TPSs. Remarkably, both AmNES/LIS enzymes were found to have relatively high *K*_m_ values for GPP and FPP compared to many other TPSs, as well as slightly higher catalytic efficiency with FPP (Nagegowda et al., 2008) suggesting that the small cytosolic GPP pool in tomato fruits is likely not sufficient to result in any detectable linalool formation in the *AmNES/LIS-1* fruits. In line with this notion, feeding of cut snapdragon flowers with [^2^H_2_] mevalolactone labeled nerolidol, however, did not show incorporation into linalool (Dudareva et al., 2005), suggesting that in snapdragon flower tissue a potentially existing cytosolic GPP pool is also too small to drive monoterpene formation.

The nerolidol emission in *AmNES/LIS-1* fruits was substantially lower (between 60-fold and almost 600-fold) than the linalool emission in *AmNES/LIS-2* fruits (Figures 2C, 3C). Remarkably, a large fraction of both terpene products, nerolidol and linalool, accumulated as glucoside derivatives in the transgenic tomato fruits (Figure 5). Since hyperaccumulation of VOCs in plant tissues was recently shown to have a detrimental effect on the integrity of cellular membranes (Adebesin et al., 2017), the observed glycosylation of the engineered terpenes might represent a mechanism to prevent autotoxicity from these compounds accumulating in the ripening transgenic fruits. In addition, glycosylated derivatives of terpenes, such as linalool, have been found in many fruits (Fan et al., 2009; Bönisch et al., 2014; Yauk et al., 2014; Wu et al., 2017) and are known to play a role in retronasal flavor perception upon fruit consumption (Parker et al., 2017). However, similar to the difference in emission of the volatile free nerolidol and linalool (Figures 2C, 3C), our analysis of these internal pools of terpene derivatives also demonstrated that the accumulation of nerolidol-glucosides in *AmNES/LIS-1* fruits was significantly lower (4- to 14-fold) than that of linalool-glucosides in *AmNES/LIS-2* fruits (Figure 5). In summary the observed differences in emission and accumulation of nerolidol and linalool in *AmNES/LIS-1* and *AmNES/LIS-2* fruits, respectively, suggest that only relatively small amounts of FPP are available for sesquiterpene formation in the cytosol, while comparatively larger amounts of GPP are available for monoterpene formation in plastids in pericarp cells of ripening tomato fruits. These results are consistent with the high activity of the plastidic MEP pathway during the ripening process of tomato fruits (Lois et al., 2000; Botella-Pavia et al., 2004), which provides precursors for the massive accumulation of carotenoids, the tetraterpene pigments (Fraser et al., 1994), and can be utilized for monoterpene formation by the introduced TPS. In contrast, the MVA pathway is highly active only during early stages of tomato fruit development (Narita and Gruissem, 1989; Jelesko et al., 1999) providing precursors for the production of sterols, which are required for the rapid cell division and expansion (Gillaspy et al., 1993). However, during the later fruit ripening stage the MVA pathway is not highly active and thus apparently provides only a limited amount of FPP that can be used for sesquiterpene formation by the introduced TPS. Similar results were obtained when two unrelated cytosolic and plastidic TPS from *Ocimum basilicum*, α-zingiberene synthase (ZIS) and geraniol synthase (GES), respectively, were expressed in tomato fruits under the control of the same ripening specific *PG* promoter (Davidovich-Rikanati et al., 2007, 2008). Although the data could not be directly compared due to the different kinetic properties of these two TPSs, the higher production of monoterpenes in *GES* fruits relative to a lower production of sesquiterpenes in *ZIS* fruits suggested a difference in the pool sizes of FPP and GPP available in the cytosol and plastids, respectively.

Moreover, a similar trend was found earlier when a number of mono- and sesquiterpene synthases were overexpressed in tobacco and Arabidopsis, and the formation of respective terpenes was analyzed in leaves. Expression of a cytosolic germacrene A synthase in Arabidopsis leaves resulted in a sesquiterpene formation that was at least 1000-fold lower compared to the monoterpene formation observed upon the expression of a plastid-targeted dual linalool/nerolidol synthase (Aharoni et al., 2003). Likewise, only trace amounts of sesquiterpenes were found upon expression of amorpha-4,11-diene synthase in tobacco leaves, while the expression of a plastid localized limonene synthase resulted in the production of significantly higher amounts of the corresponding monoterpene (Wu et al., 2006). Upon expression of a second sesquiterpene synthase, patchoulol synthase, in tobacco leaves significantly higher product levels were measured than in the amorpha-4,11-diene synthase expressing tobacco lines (Wu et al., 2006) indicating that the different kinetic properties of the introduced terpene synthases also affect the outcome. The differences in sesquiterpene and monoterpene formation that were observed when respective terpene synthases were expressed in leaves suggested the availability of only a limited pool of FPP in the cytosol and a larger pool of GPP in plastids, similar to the situation found in ripening tomato fruits. In line with these results MEP pathway genes in Arabidopsis are predominantly expressed in photosynthetic tissues, such as leaves, while MVA pathway genes are highly expressed in roots and reproductive organs containing meristematic tissue (Vranová et al., 2013).

Due to the relatively small nerolidol production in *AmNES/LIS-1* fruits and the known low activity of the MVA pathway in ripening tomato fruits (Narita and Gruissem, 1989; Jelesko et al., 1999), we tested if an increase in the metabolic flux through this pathway would result in higher sesquiterpene levels. Since HMGR is generally considered to catalyze the rate-limiting step in the MVA pathway (Hemmerlin et al., 2012), *AtHMGR1* was transiently overexpressed in ripening *AmNES/LIS-1* fruits. *AtHMGR1* expression increased nerolidol emission 5.7-fold (Figure 6A) relative to fruits injected with *Agrobacterium* carrying the empty vector control, suggesting that the metabolic flux through the MVA pathway is indeed limiting in ripening tomato fruits and provides insufficient amounts of the FPP precursor to support high levels of sesquiterpene formation. Although the plastidic MEP pathway is already quite active in ripening tomato fruits (Lois et al., 2000; Botella-Pavia et al., 2004), overexpression of the key MEP pathway enzyme DXS in tomato fruits was shown to lead to higher carotenoid levels (Enfissi et al., 2005), indicating that the metabolic flux through this pathway can be even further increased. Earlier studies have shown that cytosolic synthesis of terpenoids, including sesquiterpenes, can also be supported by precursors derived from the plastidic MEP pathway (Hemmerlin et al., 2003; Laule et al., 2003; Dudareva et al., 2005). Therefore, we tested if increased flux through the MEP pathway in ripening tomato fruits would affect cytosolic production of nerolidol and increase its level. The transient expression of *AtDXS* in *AmNES/LIS-1* fruits indeed resulted in an increased nerolidol formation relative to the empty vector control (Figure 6A), thus confirming that an enhanced metabolic flux through the MEP pathway can support cytosolic sesquiterpene formation as well. However, the 2.5-fold increase in nerolidol formation achieved by *AtDXS* co-expression in *AmNES/LIS-1* fruits was significantly lower than the 5.7-fold increase observed upon *AtHMGR1* co-expression (Figure 6A).

We utilized the same co-expression approach in ripening tomato fruits to verify if increased metabolic fluxes through the MVA and MEP pathways likewise support monoterpene formation in plastids. Transient expression of *AtHMGR1* and *AtDXS* in ripening *AmNES/LIS-2* fruits resulted in 1.8- and 3.2-fold higher linalool formation, respectively, compared to the empty vector control (Figure 6B). These results provide further evidence that the MVA and MEP pathways both in principle can provide precursors for terpene biosynthesis in the cytosol and plastids. This is consistent with previous studies demonstrating the exchange of MVA and MEP pathway derived precursors in both directions supporting production of various terpenoids in both compartments (Kasahara et al., 2002; Nagata et al., 2002; Hemmerlin et al., 2003; Laule et al., 2003; Schuhr et al., 2003; Dudareva et al., 2005). However, by comparing the extent to which increased fluxes through the MVA and MEP pathway supported nerolidol and linalool formation in *AmNES/LIS-1* and *AmNES/LIS-2* fruits, respectively (Figures 6A,B), it became evident that the pathway localized in the same compartment as the introduced terpene synthase had a more profound effect on product outcome than the pathway providing precursors via metabolic crosstalk across the plastid envelope membranes. This suggested that the yet unknown plastid envelope transporter(s) involved in this metabolite exchange potentially also represent a limiting factor for the metabolic engineering of sesquiterpenes in tomato fruits and plants in general.

Isopentenyl phosphate kinase (IPK) is a newly discovered member of the terpenoid metabolic network in plants, which is co-expressed with MVA pathway and FPPS genes (Henry et al., 2015). IPK catalyzes the conversion of IP to IPP and thus together with Nudix hydrolases regulates the ratio of these two metabolites and subsequently the metabolic flux toward prenyl diphosphate intermediates and downstream terpenoid products (Henry et al., 2015, 2018). Indeed, *AtIPK* overexpression in tobacco leaves led to up to 3.4-fold higher β-caryophyllene and 5-*epi*-aristolochene formation (Henry et al., 2015). When *AtIPK* was transiently co-expressed in *AmNES/LIS-1* tomato fruits the level of nerolidol increased by 2.9-fold relative to the empty vector control (Figure 6C), suggesting that overexpression of IPK enhances the available cytosolic FPP pool. This is likely the outcome of two processes, an increase in cytosolic IPP and DMAPP as well as an increase in FPPS activity which was shown to be inhibited by IP (Henry et al., 2015), thus further confirming the essential role of IPK in regulating and potentially enhancing the metabolic flux toward sesquiterpenes.

## Conclusion

In this study, we have taken a novel approach to evaluate the availability of prenyl diphosphate substrates for the metabolic engineering of terpene production in plants by utilizing two nearly identical bifunctional terpene synthases, *AmNES/LIS-1* and *-2*, with similar catalytic properties toward GPP and FPP, that are localized in the cytosol and plastids, respectively. The variable outcome in mono- and sesquiterpene engineering observed in our and previous studies seems to be the result of a combination of two factors: the kinetic properties of the introduced terpene synthases and the availability of the required prenyl diphosphate substrates in the respective subcellular compartment. The limited production of the sesquiterpene nerolidol observed in our experiments appears to be due to the low metabolic activity of the MVA pathway in the targeted tissue, which cannot be fully compensated by plastidic MEP pathway despite it being highly active. This conclusion was supported by the increased nerolidol formation upon co-expression of HMGR and IPK resulting in an enhanced metabolic flux through the MVA pathway toward cytosolic FPP. In contrast, increasing the metabolic flux through the MEP pathway via expression of the key pathway enzyme DXS had only a minor effect on nerolidol formation suggesting a potential limitation in the metabolic crosstalk between plastids and cytosol by the metabolite transport across the envelope membranes. Thus, future approaches toward metabolic engineering of sesquiterpene production in plants should consider the co-expression of respective TPSs with multiple key MVA pathway enzymes including HMGR and PMK (Henry et al., 2018), the central regulator IPK, as well as FPP synthase. In addition, targeted efforts toward the identification of plastid envelope transporters are required before the full potential of metabolites provided by the MEP pathway can be utilized to support engineered sesquiterpene production in the cytosol.

## Data Availability Statement

The original data presented in the study are included in the article/Supplementary Material, further inquiries can be directed to the corresponding author.

## Author Contributions

MG, EP, and ND planned and designed the research. MG, LH, SG, JL, and TN performed the experiments. MG and ND analyzed the data and wrote the manuscript with contributions from JL and EP. All authors contributed to the article and approved the submitted version.

## Conflict of Interest

The authors declare that the research was conducted in the absence of any commercial or financial relationships that could be construed as a potential conflict of interest.

## Supplementary Material

Supplementary Figure 1 Metabolic profiling of transgenic tomato lines overexpressing *AmNES/LIS-1* and *AmNES/LIS-2* under control of the *PG* promoter. Emission of the sesquiterpene nerolidol **(A)** and the monoterpene linalool **(B)** from ripe tomato fruits (*AmNES/LIS-1* line B5 and *AmNES/LIS-2* line U8) were analyzed by GC-MS. IS, internal standard.

Supplementary Figure 2 Mass spectra of two unknown terpene compounds observed in the internal pools extracted from *AmNES/LIS-1* fruits (see Figure 4A). Extracts were analyzed by GC-MS and mass spectra are shown for: **(A)** unknown terpene 1; **(B)** unknown terpene 2.

Supplementary Table 1 Primers for genotyping and qRT-PCR analyses of transgenic tomato lines.
