# Supplementary figures and images for: Overcoming Bottlenecks for Metabolic Engineering of Sesquiterpene Production in Tomato Fruits
Source: Front Plant Sci. 2021 Jun 17;12:691754. doi: 10.3389/fpls.2021.691754 (PMC8248349; doi:10.3389/fpls.2021.691754)

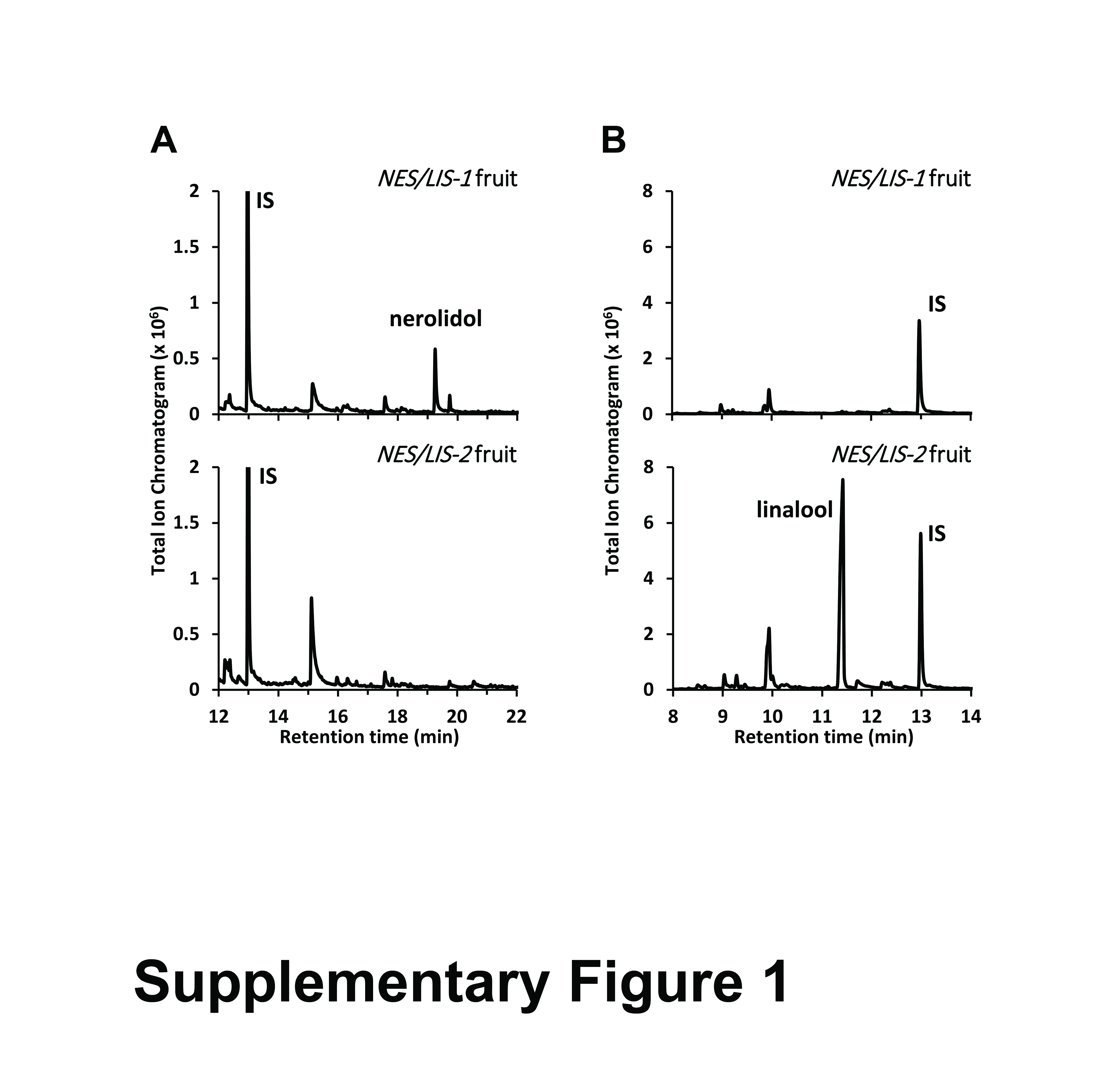

Supplement: Supplementary Figure 1 — Metabolic profiling of transgenic tomato lines overexpressing AmNES/LIS-1 and AmNES/LIS-2 under control of the PG promoter. Emission of the sesquiterpene nerolidol (A) and the monoterpene linalool (B) from ripe tomato fruits (AmNES/LIS-1 line B5 and AmNES/LIS-2 line U8) were analyzed by GC-MS. IS, internal standard. [file Image_1.jpg]

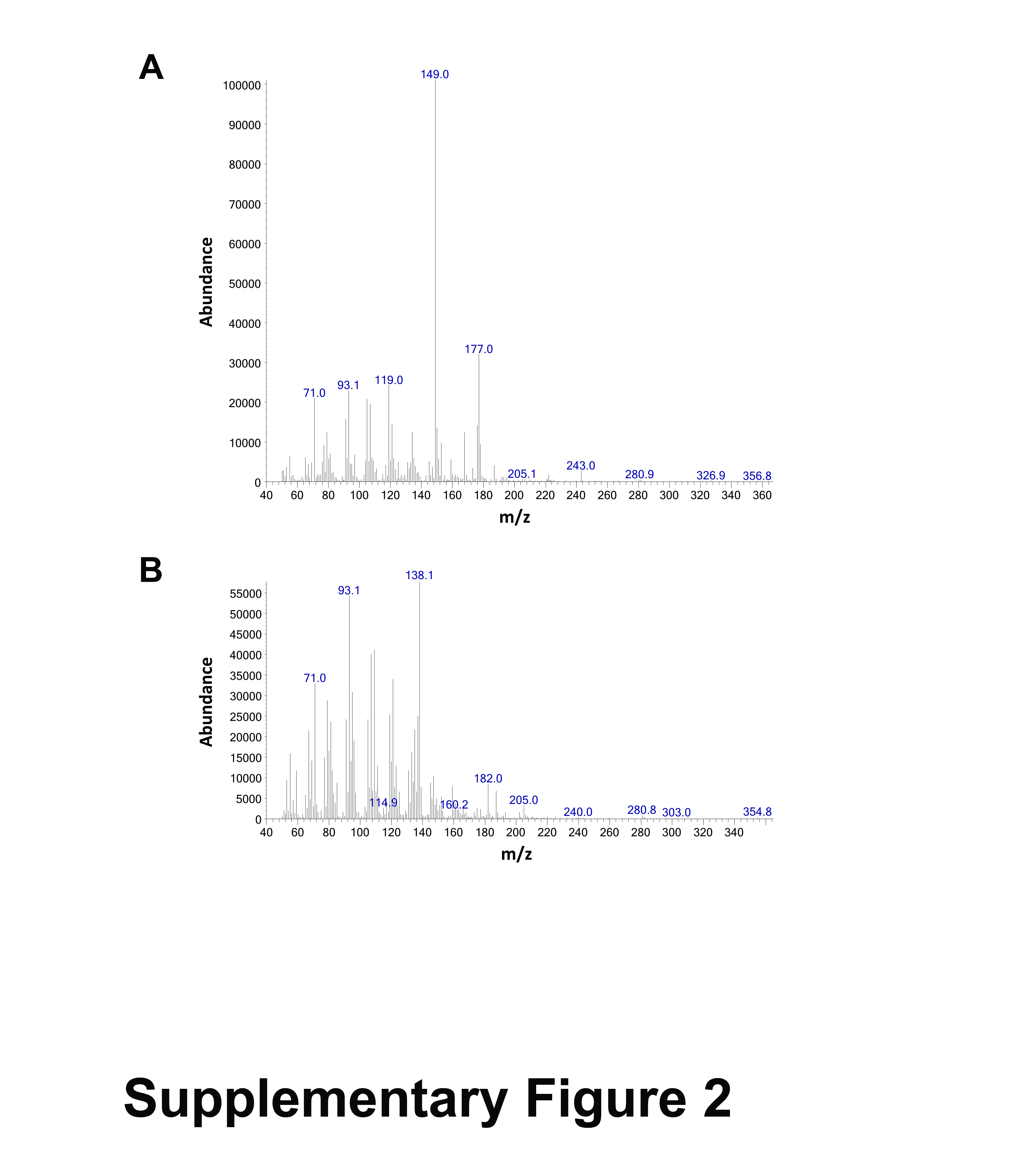

Supplement: Supplementary Figure 2 — Mass spectra of two unknown terpene compounds observed in the internal pools extracted from AmNES/LIS-1 fruits (see Figure 4A). Extracts were analyzed by GC-MS and mass spectra are shown for: (A) unknown terpene 1; (B) unknown terpene 2. [file Image_2.jpg]
