# Supplementary material for: Overcoming Bottlenecks for Metabolic Engineering of Sesquiterpene Production in Tomato Fruits
Source: Front Plant Sci. 2021 Jun 17;12:691754. doi: 10.3389/fpls.2021.691754 (PMC8248349; doi:10.3389/fpls.2021.691754)
Supplement: Supplementary Table 1 — Primers for genotyping and qRT-PCR analyses of transgenic tomato lines. [file Table_1.DOCX]

**SUPPLEMENTARY TABLE 1**

Primers for genotyping and qRT-PCR analyses of transgenic tomato lines

| **Primer** | **Sequence** |
| --- | --- |
| *LIS1&2* for | 5´-ATGAGTAACCTTCATGTAAAGA-3´ |
| *LIS1* rev | 5´-CTAATCAAACAACATAATCTTA-3´ |
| *LIS2* rev | 5´-CTAATCAAACAACATAAACTTA-3´ |
| *LIS1&2* qRT for | 5´-GTCTCGCAAGAAACCAACCATT-3´ |
| *LIS1&2* qRT rev | 5´- GCTCGATTCTTTGCTCCGATA-3´ |
